# Mass type-specific sparse representation for mass classification in computer-aided detection on mammograms

**DOI:** 10.1186/1475-925X-12-S1-S3

**Published:** 2013-12-09

**Authors:** Dae Hoe Kim, Seung Hyun Lee, Yong Man Ro

**Affiliations:** 1 Image and Video Systems Lab, Dept. of Electrical Engineering, Korea Advanced Institute of Science and Technology (KAIST), Yuseong-Gu, Daejeon, 305-701, Republic of Korea

## Abstract

**Background:**

Breast cancer is the leading cause of both incidence and mortality in women population. For this reason, much research effort has been devoted to develop Computer-Aided Detection (CAD) systems for early detection of the breast cancers on mammograms. In this paper, we propose a new and novel dictionary configuration underpinning sparse representation based classification (SRC). The key idea of the proposed algorithm is to improve the sparsity in terms of mass margins for the purpose of improving classification performance in CAD systems.

**Methods:**

The aim of the proposed SRC framework is to construct separate dictionaries according to the types of mass margins. The underlying idea behind our method is that the separated dictionaries can enhance the sparsity of mass class (true-positive), leading to an improved performance for differentiating mammographic masses from normal tissues (false-positive). When a mass sample is given for classification, the sparse solutions based on corresponding dictionaries are separately solved and combined at score level. Experiments have been performed on both database (DB) named as Digital Database for Screening Mammography (DDSM) and clinical Full Field Digital Mammogram (FFDM) DBs. In our experiments, sparsity concentration in the true class (SCTC) and area under the Receiver operating characteristic (ROC) curve (AUC) were measured for the comparison between the proposed method and a conventional single dictionary based approach. In addition, a support vector machine (SVM) was used for comparing our method with state-of-the-arts classifier extensively used for mass classification.

**Results:**

Comparing with the conventional single dictionary configuration, the proposed approach is able to improve SCTC of up to 13.9% and 23.6% on DDSM and FFDM DBs, respectively. Moreover, the proposed method is able to improve AUC with 8.2% and 22.1% on DDSM and FFDM DBs, respectively. Comparing to SVM classifier, the proposed method improves AUC with 2.9% and 11.6% on DDSM and FFDM DBs, respectively.

**Conclusions:**

The proposed dictionary configuration is found to well improve the sparsity of dictionaries, resulting in an enhanced classification performance. Moreover, the results show that the proposed method is better than conventional SVM classifier for classifying breast masses subject to various margins from normal tissues.

## Background

According to the World Health Organization, breast cancer is the major leading cause of both incidence and mortality in women [1]. It has been generally believed that screening mammography is the most cost-effective approach for early detection of breast cancer [2]. For this reason, considerable research efforts have been devoted to develop Computer-Aided Detection (CAD) systems, which would be beneficial for detecting breast lesions.

In practical CAD systems, it is generally difficult to achieve high sensitivity at a low false positive (FP) detection rate [3]. Due to the variability of mass margins and the inherent superposition of normal tissues in mammography, mammographic mass detection can be much more challenging compared to micro-calcification detection [4]. In particular, a high number of FP detections could induce unnecessary breast biopsies so that patients would get anxious and unnecessary costs expense. Thus, reducing the number of FP detections is of great importance in practical breast cancer screening based on mammography.

In recent years, Sparse Representation based Classification (SRC) [5] has been increasingly important in the field of signal processing. The objective of sparse representation is to represent a signal pattern in a compact and sparse way for the purpose of representing a signal pattern with a few numbers of atoms [5]. Referring to [5], high degree of sparsity can be desirable to improve classification performance as much as possible. Generally, a higher sparsity could be achieved if a fewer number of atoms is able to represent signal patterns. Sparse representation could contain discriminating and crucial information of a signal pattern. In light of this fact, SRC may be appropriate to capture the unique and apparent patterns present in breast masses. Thus, it is reasonable to assume that applying SRC to mammographic CAD system can improve classification performance.

A solid and well-established study on the use of SRC for classification applications has been well-documented in the research area of face recognition. Wright et al. [6] demonstrated that SRC was robust to face occlusion and they showed that SRC outperformed other face recognition algorithms when classifying corrupted face images. However, only few studies proposed the use of SRC for developing classification algorithms devised for CAD systems. Liu et al. [7] designed a CAD system utilizing SRC with learned dictionaries in classifying lesions of colon and lung. Herrndsvela [8] made use of SR as pixel-wise classification to determine whether each pixel is located in mass regions or not. However, this paper has been limited to only deal with one type of possible mass margins (i.e., circumscribed mass). In addition, the feature for classifying pixels was limited to image intensities of *n *by *n *neighbourhood of each pixel. However, image level information is likely to be more affected by breast densities or surrounding tissues structures, mainly due to the direct use of pixel values.

The margin of a mass (i.e., the border of a mass) should be carefully examined because it is one of the most important criteria in determining whether the mass is benign or malignant [4]. Radiologists classify the mass margins into the following five types [4]: circumscribed, obscured, micro-lobulated, ill-defined, and spiculated margins. In most studies on SRC-CAD, breast masses are treated as a single class. However, this approach causes the increased diversity in positive class and subsequently degrades *sparsity *in sparse representation.

To cope with above-mentioned problem, we propose a dictionary configuration framework designed for improving the *sparsity *in terms of mass margins. The proposed dictionary configuration is incorporated into the sparse representation based classification (SRC) for mammographic mass classification in CAD systems. To this end, we adopt divide and conquer strategy [9] on the mass classification with various margins. In the proposed dictionary configuration, we construct *individual and separate dictionaries each corresponding to a particular type of mass margins commonly encountered in clinical screening process*. Thus, the number of dictionaries is equal to the number of types of mass margins predefined. The sparse solutions- each of which is solved using a corresponding dictionary component- are effectively combined using a score level fusion to make the final decision. In addition, our proposed method has been designed by adopting a dictionary learning in order to overcome insufficient sample problem. Further, the classification is performed at feature level rather than at image level in order to effectively make use of relevant information of mass margins in a better way and to reduce data dimension and computational cost [6].

Experiments had been conducted using the public DDSM database [10] and the clinical mammography dataset provided from a hospital in order to test the effectiveness of the proposed framework on mammograms. Experimental results show that the proposed method is able to achieve high sensitivity at a low FP rate compared with a well-established and generally used support vector machine (SVM) classifier in mammographic CAD systems.

The rest of this paper is organized as follows. In Section "*Methods*", we briefly introduce the region-of-interest (ROI) segmentation and feature extraction method used in this paper. In sequence, the proposed dictionary configuration and the sparse representation based classification (SRC) are described in detail. In Section "Results and discussion", experimental results and discussion are presented. The conclusion is drawn in Section "Conclusion".

## Methods

### ROI segmentation and feature extraction

**Figure 1:**
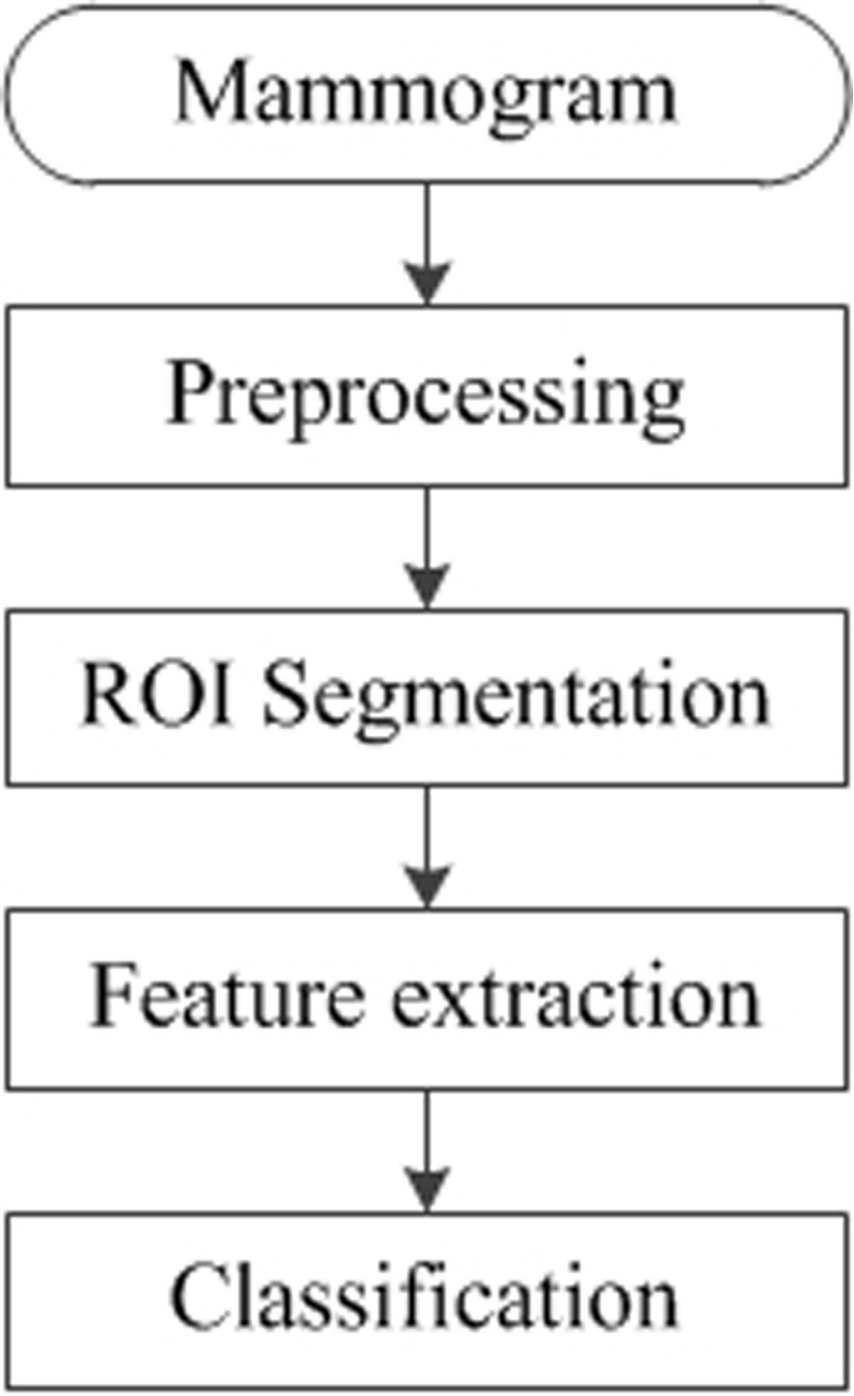
**Generic framework of mammographic Computer-Aided Detection (CAD) algorithms**.

Referring to [11], mammographic CAD systems generally consist of the following four stages: image preprocessing (enhancement), ROI segmentation, feature extraction, and classification as described in Figure 1. The focus of this paper is to develop the effective classification method so as to increase the mass classification performance. Since ROI segmentation and feature extraction are prerequisite steps prior to performing classification of ROIs, we briefly describe the segmentation and feature extraction technique used in this paper.

**Figure 2:**
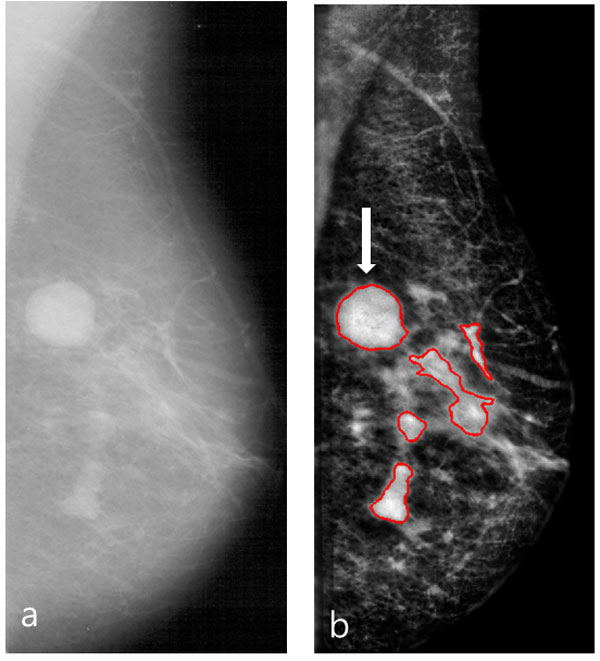
**An example of the enhanced mammogram and segmented ROIs**. (a) A mammogram from DDSM DB. (b) An enhanced mammogram with segmented ROIs, while the white colored arrow indicates a true mass.

For image preprocessing, the mass enhancement technique [12] (developed by our group) is applied to original mammogram images for the purpose of increasing mass detection sensitivity. In addition, the multi-level thresholding based mass segmentation algorithm proposed in [13] is used to detect and segment mass candidates (ROIs) from the enhanced mammogram. Figure 2 shows an example of an enhanced mammogram with segmented ROIs generated by the preprocessing and ROI segmentation. As shown in the Figure 2, the preprocessing effectively increases the contrast of mammogram and ROI segmentation well detects and segments mass ROIs. The segmented ROIs were used as input for feature extraction. Herein, we used four different feature subspaces: texture, shape, intensity, and spiculation features. The features used in our study are summarized in Table 1. The features listed in Table 1 were used as a particular feature representation during the generation of dictionaries in the proposed SRC framework.

**Table 1:** Description for the features used in the proposed SRC framework

Type	Features	NF
Texture	**Local binary pattern (LBP) **[23-25] Uniform LBP histograms are computed from the segmented object; LBP operator with a circularly symmetric neighbourhood of P members on a circle radius of R is employed; the three-resolution combination is used by setting LBP parameters (P,R) values of (8,1), (8,2), and (8,3)	354
	
	**Spatial gray level dependence (SGLD) **[26] 13 features, namely, "correlation", "energy", "entropy", "inertia", "inverse difference moment", "sum average", "sum variance", "sum entropy", "difference energy", "difference variance", "difference entropy", "information measure of correlation 1", "information measure of correlation 2" are extracted from each SGLD matrix at six different inter-pixel distances (*d *= 1, 2, 4, 6, 8, and 10) and in four directions ($\theta =0^{\circ },45^{\circ },90^{\circ },$and $135^{\circ }$), are used to calculate 24 SGLD matrices, yielding 312 SGLD features	312
	
	**Run length statistics (RLS) **[27] Five features, namely, "short run emphasis", "long runs emphasis", "gray-level nonuniformity", "run-length nonuniformity", and "run percentage" are obtained from the gray level run length matrices with four directions, $\theta =\left\{0^{\circ },45^{\circ },90^{\circ },135^{\circ }\right\}$	20
	
	**Gray level difference statistics (GLDS) **[28] Four features "contrast", "angular second moment", "entropy", and "mean" are extracted from the gray level difference statistics vector; six different inter-pixel distances (*d *= 1, 2, 4, 6, 8, and 10) and four directions ($\theta =0^{\circ },45^{\circ },90^{\circ },$and $135^{\circ }$) are used to calculate 24 GLDS vectors, yielding 96 GLDS features	96

Shape	**Normalized radial length (NRL) **[29] NRL mean, NRL standard deviation, NRL area ratio, NRL zero crossing count, NRL entropy	5

Intensity [11]	**Contrast measure, Average gray level, Standard deviation, Skewness, Kurtosis**	5

Spiculation	**Region-based stellate features **[30] Means of pixel-wise stellate features are computed from the three local regions (core, inner, and outer regions, respectively); standard deviation of means of pixel-wise stellate features are computed from the three local regions; differences of means of pixel-wise stellate features are computed from the three local regions	20

NF is abbreviation of number of features.

### Classification of breast masses using the proposed method

#### 1. Sparse representation based classification

In this section, we first briefly review a SRC algorithm and describe the way of applying SRC algorithm for classification of segmented ROIs. Note that all of the features described in Table 1 are used to find the corresponding sparse representations of segmented ROIs and to perform the classification task.

To formulate the classification problem based on sparse representation, mammographic mass features are used as atoms of dictionaries. *n_i _*training feature vectors from the *i^th ^*class are put together into a dictionary of the *i^th ^*class as $A_{i}=\left[v_{i,1},v_{i,2},\cdots \;,v_{i,n_{i}}\right]\in R^{d\times n_{i}}$, where *d *is the feature dimension and *n_i _*is the number of samples in the *i^th ^*class. Note that, in the present work, we are performing a binary classification task; thus, *i *= *mass *and *normal*, representing breast masses (positive class) and normal tissues (negative class). By concatenating feature vectors from the mass and normal tissue training samples, a dictionary is generated as $A=\left[A_{mass},A_{normal}\right]$. When classifying a test sample, the test feature vector $y\in R^{d}$ can be approximated as a linear combination of the training feature vectors from corresponding class *i*. Since the membership to the *i^th ^*class of the test feature vector is initially unknown, the linear combination of **y **can be rewritten as follows using the dictionary **A**:

(1) $$y=Ax_{0},$$

where $x_{0}={\left[0,\cdots \;,0,\alpha_{i,1},\alpha_{i,2},\cdots \;,\alpha_{i,n_{i}},0,\cdots \;,0\right]}^{T}\in R^{n}$ is a coefficient vector whose entries are zero except those belonging to the corresponding the *i^th ^*class.

Since a valid test sample **y **is likely to be sufficiently represented using only the training samples from the same class, it is possible to find a sparse solution of Eq. (1) by solving the following $\ell^{0}$-minimization problem [6]:

(2) $$\hat{x}=\arg\text{min}||x|{|}_{0}\;\text{subject to }Ax=y,$$

where $||\cdot |{|}_{0}$ denotes the $\ell^{0}$-norm, which counts the number of nonzero entries in a input vector.

However, the $\ell^{0}$-minimization problem is NP-hard (Non-deterministic Polynomial-time hard). Donoho [14] proved that the solution of the $\ell^{0}$-minimization can be approximated to that of $\ell^{1}$-minimization. Therefore, Eq. (2) can be rewritten as [6]

(3) $$\hat{x}=\text{arg}\underset{x}{\text{min}}||x|{|}_{1}\;\text{subject to}\;||Ax-y|{|}_{2}\leq \varepsilon .$$

Then, we compute residuals for each class as follows:

(4) $$r_{i}\left(y\right)=||y-A\delta_{i}\left(\hat{x}\right)|{|}_{2},\;\text{for }i=mass\;\text{and }normal,$$

where $\delta_{i}$ is the characteristic function which selects the coefficients associated with the *i^th ^*class.

Note that small residual means test feature vector is sufficiently approximated as a linear combination of the training feature vectors from corresponding class. Therefore, the test feature vector **y **can be classified to the class that minimizes the residual:

(5) $$\text{identity }\left(y\right)=\text{arg}\underset{i}{\text{min}}r_{i}\left(y\right).$$

#### 2. The proposed dictionary configuration

In this section, we explain the proposed dictionary configuration method. For this purpose, we first describe the dictionary learning method adopted in this paper. Generally, dictionary generation can be categorized into two approaches: the analytic approach (i.e., wavelets) and the learning-based approach (i.e., K-SVD, FDDL). Advantages of the learning-based approach are the much finer-tuned (i.e., more sophisticated) dictionaries they produce compared to the analytic approaches, and their significantly better performance in applications [15]. It should be pointed out that mammographic mass classification is generally quite difficult due to the large variability in the appearance of mass patterns [4] such as its irregular size, obscured borders, and complex mixtures of margin types. Therefore, the learning-based dictionary generation is more appropriate for constructing dictionary that aims at maximizing mass classification performance, thanks to their capability of characterizing a wide variety of mammographic mass patterns in a sophisticated way.

In typical mammographic CAD design, the number of positive training samples may be often insufficient because the training samples should be divided into small subsets according to its type of margin. However, it should be noted that to correctly classifying a large variety of mass types found in clinical practices, it would be desirable that dictionaries should contain a sufficient number of mass samples for each mass type to achieve better classification performances of SRC [16]. Also note that the goal of using sparse representation in our method is to express a given mass example as linear combination of a small number of atoms taken from a "dictionary" resource. Hence, large-sized dictionaries may lead to a better sparse solution than small-sized dictionary [17,18]. In order to effectively represent mass examples with a given atoms, the Fisher discrimination dictionary learning (FDDL) [18] has been incorporated into the proposed dictionary configuration method. The FDDL aims to learn a structured dictionary whose sub-dictionaries have specific class labels. Each sub-dictionary of the learned whole dictionary has good representation power to the samples from the corresponding class, but has poor representation power to the samples from other classes [18]. The FDDL iteratively updates the dictionary so that the learned dictionary would have smaller within-class scatter degree while maintaining larger between-class scatter degree, resulting in improved SRC performances.

**Figure 3:**
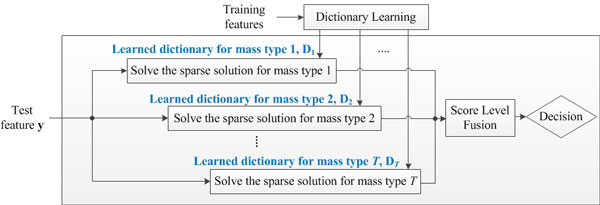
**Proposed dictionary configuration method description**. Note that the proposed dictionary configuration has been performed at the classification stage shown in Figure 1.

In general mass classification task, suspicious regions are classified as mass or normal tissues, i.e., binary classification problem. Herein, we assume that true masses are assigned to positive class while normal tissues for negative class. However, this results in increasing the diversity in positive class, and degrades sparsity in sparse representation. To cope with the problem, we propose a dictionary configuration framework that improves sparsity in terms of mass margins within conventional SRC framework for CAD systems. Note that as shown in Figure 3, the proposed dictionary configuration is used at the classification stage. Key property of the proposed dictionary configuration is to increase the sparsity of each dictionary, because each dictionary contains positive samples that have the similar margin characteristics. In the proposed SRC framework, *T *dictionaries are learned separately where *T *is the number of types of mass margins. It should be noted that each dictionary contains features from mass samples in a single type of mass margins and features from normal tissues.

In addition, one major problem of typical CAD systems is the large number of false positives. Hence, an organized dictionary is likely to be unbalanced due to the difference in the number between true-positive and false-positive samples. This would make the sparse solution based on that dictionary to be highly biased toward the class that contain a large number of samples. Consequently, this biased sparse solution could cause low true positive rate and high true negative rate within a SRC framework. To address the aforementioned issue, the proposed SRC framework is designed for performing *random sampling *on negative samples, aiming to make the balanced dictionary.

After constructing dictionaries, the FDDL algorithm [18] is separately applied to individual dictionary; this can improve the *sparsity *of each dictionary. We now explain how to obtain the sparse solution for each type of mass margins. Let us denote the learned dictionary by **D***_t_*, where *t *is an index of mass margin types and *t *= 1,..., *T*, and *T *is the total number of types of mass margins. When given **D***_t _*and a test feature vector **y**, sparse solutions of the test feature vector **y **for each **D***_t _*can be solved by using Eq. (3). Without loss of generality, the sparse solution for each type of mass margins can be defined as follows:

(6) $${\hat{x}}_{t}=\text{arg}\underset{x}{\text{min}}||x|{|}_{1}\;\text{subject to }||D_{t}x-y|{|}_{2}\leq \varepsilon_{t}\;\text{for }t=1,...,T.$$

The residuals of sparse solutions derived from each dictionary according to the types of mass margins in Eq. (4) are fused at score level by calculating residual corresponding to mass and normal classes as follows:

(7) $$\text{Re}{\text{s}}_{i}=\overset{T}{\underset{t=1}{\sum }}{∥y-D_{t}\delta_{i}\left({\hat{x}}_{t}\right)∥}_{2}\;\text{for }i=mass\text{ or }normal.$$

Note that in Eq. (7), the fused residual represents reconstruction error with the given class *i*. Therefore, the fused residual is utilized as final decision. Smaller residual indicates that the test sample is sufficiently approximated with the training samples from corresponding class. Thus, the test sample can be classified to the class that achieves the minimization of the residual. In detail, in case of a normal ROI, residuals of the normal class should be smaller than that of the mass class for all dictionaries. Therefore, the fused residual also have a smaller fused residual for the normal class. In case of a mass ROI, a residual of the mass class should small compared to that of the normal class in the corresponding margin-type dictionary. Therefore, the fused residual of mass class should have a smaller values compared to that of normal class.

### Experimental setup

**Figure 4:**
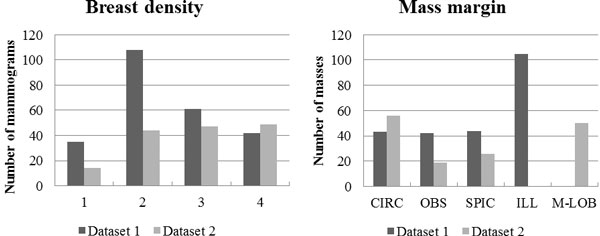
**Statistical information of the datasets on Dataset 1 and Dataset 2**. Distribution of breast densities (left) and mass margins (right), CIRC: circumscribed, OBS: obscured, SPIC: spiculated, ILL: ill-defined, M-LOB: micro-lobulated.

The proposed dictionary configuration based classification method was tested on both public data, so-called Digital Database for Screening Mammography (DDSM) [10], and the real clinical dataset provided from Samsung Medical Center (SMC). From DDSM DB, we collected 303 mammograms (each with one mass) containing benign or malignant masses; it will be referred to as the "Dataset 1". The second dataset consists of a total of 165 clinical mammograms (containing benign or malignant masses). We called this DB as the "Dataset 2". Figure 4 shows information of Dataset 1 and Dataset 2, respectively, in terms of mass margin and breast density characteristics. It can be seen from Figure 4 that the masses with different margins and densities found in clinical practice were well represented in the used datasets by containing a variety of mass margins and breast densities commonly encountered in clinical mammographic CAD systems. In addition, it is known that it is hard to detect and classify masses in high density breast, because masses are concealed by surrounding Parenchyma [19]. As shown in the statistics, we tested mass ROIs with dense tissue to cover samples those are hard to classify.

By using the segmentation method described in "Methods" section, a total of 2,725 ROIs (234 masses and 2,491 normal tissues) and 691 ROIs (151 masses and 540 normal tissues) were automatically generated by using Dataset 1 and Dataset 2, respectively. The DDSM provides annotations of the true masses presented in each image [10], while for each clinical mammogram (coming from SMC), the region of interest containing the mass was annotated by a Mammography Quality Standards Act-approved radiologist. These annotations were considered as the ground truth in our experiments. Using ground truth information, a generated ROI was considered as a true mass only if it met the following two criteria [20]: (1) the centroid of a segmented region is included in the annotated area, and (2) a segmented region intersects with the true mass region more than 25%.

Evaluation protocol used in this paper was designed based on 10-fold cross validation scheme, i.e., a portion of 90% mass and normal tissue ROIs were used for training samples to construct dictionaries, while the rest of 10% mass and normal tissue ROIs were used for testing samples. To guarantee stable classification results, 30 independent runs of 10-fold cross validation were executed. Thus, all of the results reported were averaged over 30 runs with 10-fold cross validation.

To objectively quantify the improvement of sparsity compared to the conventional *single dictionary configuration *that contains various mass margins into a single dictionary, sparsity concentration in true class (SCTC) is defined as follows:

(8) $$\text{SCTC}\left(\hat{x}\right)=\frac{||\delta_{true}\left(\hat{x}\right)|{|}_{1}}{||\hat{x}|{|}_{1}}\in \left[0,1\right],$$

where $\delta_{true}$ is the characteristic function that selects the sparse coefficients associated with the true class of a test sample **y **and ${\hat{x}}_{t}$ represents the sparse solution for each type of mass margins (see Eq. (6) for definition).

In order to evaluate the classification performance of the proposed SRC framework, area under the receiver operating characteristic (ROC) curve [21] was used (denoted by AUC) because AUC is a commonly used performance index for evaluating classification algorithms developed for mammographic CAD applications [11]. To evaluate the ROC curve for the proposed method, the difference between Res*_mass _*and Res*_normal _*is used as a confidence value because if a test sample has higher residual to mass class compared to normal class, it is reasonable to assume that the sample is much similar to the mass class. For comparative purpose, a state-of-the-art support vector machine (SVM) classifier [22] that utilizes a radial basis function kernel was employed.

## Results and discussion

Table 2 shows the value of SCTCs (defined in Eq. (8)) of each mass margin using the conventional single dictionary configuration and the proposed dictionary configuration. Note that the SCTC value of each mass margin was computed when the corresponding mass margin was used as a test sample. Also note that the values of SCTCs in Table 2 have been averaged over 30 runs. The experimental results indicate that the proposed dictionary configuration is found to work well in terms of improving the sparsity of dictionary. Especially, the proposed method improved SCTC of up to 13.9% and 23.6% on Dataset 1 and Dataset 2 respectively. Table 3 shows the values of AUC for both the single dictionary configuration and the propose dictionary configuration. As shown in the Table 3, the proposed dictionary configuration attains considerably better AUC compared to the single dictionary configuration. This result indicates that the improved sparsity would have a positive influence on the classification performance. Also, it can be seen that in Table 4, the proposed method considerably outperforms the SVM classifier, where the proposed method is able to increase classification performance with 8.2% and 22.1% (in terms of AUC values) on Dataset 1 and Dataset 2, respectively, compared to the SVM classifier. These results validates that the proposed method has high potential for reducing false-positive detections in mammographic CAD systems.

**Table 2:** Comparisons of SCTC of each mass margin between the single and proposed dictionary configuration

		Mass margins				
		
Dataset	Dictionary configuration	Ill-defined	Micro-lobulated	Circumscribed	Spiculated	Obscured
Dataset 1	Single	0.5610	N/A	0.5570	0.5918	0.5478
		
	Proposed	0.5947	N/A	0.5942	0.5938	0.5473

Dataset 2	Single	N/A	0.5123	0.5079	0.4966	0.4722
		
	Proposed	N/A	0.5818	0.5362	0.5146	0.5839

N/A means the dataset originally does not contains the corresponding mass margin type.

**Table 3:** Comparisons of AUC obtained using the proposed dictionary configuration versus the single dictionary configuration

Dataset	Classification method	Averaged AUC
Dataset 1	SRC framework with the single dictionary configuration	0.7751
	
	SRC framework with the proposed dictionary configuration	0.8392

Dataset 2	SRC framework with the single dictionary configuration	0.6591
	
	SRC framework with the proposed dictionary configuration	0.8047

**Table 4:** Comparisons of AUC between the SVM and proposed dictionary configuration

Dataset	Classification method	Averaged AUC
Dataset 1	SVM	0.8155
	
	SRC framework with the proposed dictionary configuration	0.8392

Dataset 2	SVM	0.7211
	
	SRC framework with the proposed dictionary configuration	0.8047

**Figure 5:**
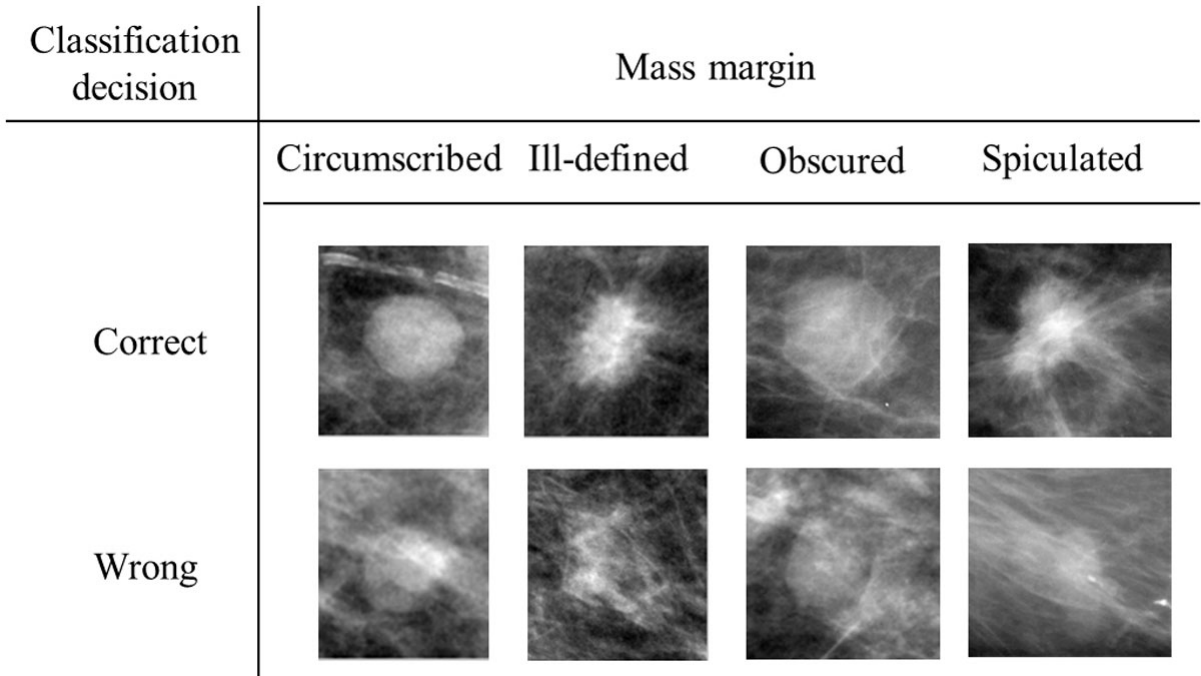
**Examples of correctly and incorrectly classified mass ROIs**. The correctly and incorrectly classified ROIs were selected among ROIs those are correctly and incorrectly classified during all of 30 runs, respectively.

Figure 5 shows examples of correctly and incorrectly classified mass ROIs. As shown in the Figure 5, correctly classified mass ROIs have more clear hyper-dense core regions and differentiable with surrounding tissues compared to incorrectly classified mass ROIs. The result indicates a weakness of the proposed method that mass ROIs should have apparent characteristics compared to surrounding tissues. Moreover, it should be noted that correctly classified mass ROIs have many number of similar samples. It indicates that to correctly classify the incorrectly classified mass ROIs, training samples should have more samples those have similar characteristics to the incorrectly classified mass ROIs.

## Conclusions

In this paper, we propose a new sparse representation based classification (SRC) algorithm based on so-called mass type-specific dictionary configuration for mammographic CAD systems. It has been found that the proposed method is beneficial for improving mass type-dependent sparsity. In addition, experimental result validate that the proposed dictionary configuration algorithm can improve the sparsity of dictionary, thus leading to increased classification performance. Furthermore, experimental results show that the proposed method is considerably better than the conventional SVM classifier (extensively used for classification applications in CAD systems of breast masses on mammography) for differentiating mammographic masses (confined to various margins) from normal tissues.

For further work, information fusion (e.g., a complementary design) from different levels (i.e., image level and feature level) should be investigated to get the better classification performances.

## List of abbreviations

CAD: Computer-Aided detection; SRC: Sparse representation based classification; FDDL: Fisher discrimination dictionary learning; DDSM: Digital database for screening mammography; FFDM: Full field digital mammogram; SCTC: Sparsity concentration in the true class; ROC: Receiver operating characteristic; AUC: Area under the ROC curve; FP: False positive; SVM: Support vector machine; ROI: Region-of-interest; LBP: Local binary pattern; SGLD: Spatial gray level dependence; RLS: Run length statistics; GLDS: Gray level difference statistics; NRL: Normalized radial length; NP-hard: Non-deterministic polynomial-time hard.

## Competing interests

The authors declare that they have no competing interests.

## Authors' contributions

DHK developed the preprocessing enhancement and ROI segmentation, feature extraction algorithms, dictionary learning algorithm and SVM classifier, participated in the study design and coordination, drafted, revised, and finalized the manuscript, analyzed the results, guided SHL in how to evaluate the classification performances. SHL worked on the algorithm design, developed SRC framework, conducted experiments, and drafted the manuscript. YMR contributed to discussion and suggestions throughout this topic, and revised and finalized the manuscript. All authors read and approved the final manuscript.

## Authors' information

DHK received the B.S. degree from Hanyang University, Seoul, Korea, in 2010, and the M.S. degree from the Korea Advanced Institute of Science and Technology (KAIST), Daejeon, Korea, in 2012. He is currently pursuing the Ph.D. degree with the Image and Video Systems Lab., Department of Electrical Engineering, KAIST. His research interests are image preprocessing, object segmentation and medical image processing. SHL received the B.S. degree from Korea University, Seoul, Korea, in 2011, and the M.S. degree from KAIST, Daejeon, Korea, in 2013. YMR received the B.S. degree from Yonsei University, Seoul, Korea, and the M.S. and Ph.D. degrees from the Korea Advanced Institute of Science and Technology (KAIST), Daejeon, Korea. He is currently a Full Professor with the Department of Electrical Engineering, KAIST, where he directs the Image and Video Systems Laboratory. In 1987, he was a Visiting Researcher with Columbia University, New York, and from 1992 to 1995, he was a Visiting Researcher with the University of California, Irvine, and KAIST. He was a Research Fellow with the University of California, Berkeley, and a Visiting Professor with the University of Toronto, Toronto, ON, Canada, in 1996 and 2007, respectively.

## References

[B1] Boyle P Levin B World cancer report 2008 IARC Press, International Agency for Research on Cancer

[B2] Freer TW Ulissey MJ Screening mammography with computer-aided detection: Prospective study of 12,860 patients in a community breast center Radiology 2001 12 781 786 11526282 10.1148/radiol.2203001282

[B3] Nishikawa RM Current status and future directions of computer-aided diagnosis in mammography Computerized Medical Imaging and Graphics 2007 12 224 235 17386998 10.1016/j.compmedimag.2007.02.009

[B4] Heywang-Köbrunner S Schreer I Diagnostic breast imaging 2001 Thieme

[B5] Huang K Aviyente S Sparse representation for signal classification Advances in neural information processing systems 2006 609 616

[B6] Wright J Yang AY Ganesh A Sastry SS Ma Y Robust face recognition via sparse representation Pattern Analysis and Machine Intelligence, IEEE Transactions on 2009 12 210 227 10.1109/TPAMI.2008.79 19110489

[B7] Liu M Lu L Ye X Yu S Salganicoff M Sparse classification for computer aided diagnosis using learned dictionaries Medical Image Computing and Computer-Assisted Intervention (MICCAI) 2011 Springer 41 48 10.1007/978-3-642-23626-6_6 22003682

[B8] Herredsvela J Engan K Gulsrud TO Skretting K Detection of masses in mammograms by watershed segmentation and sparse representasions using learned dictionaries Proc of NORSIG 2005

[B9] Duda RO Hart PE Stork DG Pattern classification 2012 Wiley-interscience

[B10] Heath M Bowyer K Kopans D Moore R Kegelmeyer P The digital database for screening mammography Proceedings of the 5th international workshop on digital mammography 2000 212 218

[B11] Cheng H Shi X Min R Hu L Cai X Du H Approaches for automated detection and classification of masses in mammograms Pattern recognition 2006 12 646 668

[B12] Kim DH Choi JY Choi SH Ro YM Mammographic enhancement with combining local statistical measures and sliding band filter for improved mass segmentation in mammograms SPIE Medical Imaging 2012 83151Z 83156

[B13] Hong B-W Brady M A topographic representation for mammogram segmentation Medical Image Computing and Computer-Assisted Intervention (MICCAI) 2003 Springer 730 737

[B14] Donoho DL For most large underdetermined systems of linear equations the minimal Communications on pure and applied mathematics 2006 12 797 829

[B15] Rubinstein R Zibulevsky M Elad M Double sparsity: Learning sparse dictionaries for sparse signal approximation Signal Processing, IEEE Transactions on 2010 12 1553 1564

[B16] Olshausen BA Field DJ Sparse coding with an overcomplete basis set: A strategy employed by V1? Vision research 1997 12 3311 3325 9425546 10.1016/s0042-6989(97)00169-7

[B17] Jiang Z Lin Z Davis LS Learning a discriminative dictionary for sparse coding via label consistent k-svd Computer Vision and Pattern Recognition (CVPR), 2011 IEEE Conference on 2011 IEEE 1697 1704

[B18] Yang M Zhang L Feng X Zhang D Fisher discrimination dictionary learning for sparse representation Computer Vision (ICCV), IEEE International Conference on 2011 IEEE 543 550

[B19] Chan H-P Wei D Helvie MA Sahiner B Adler DD Goodsitt MM Petrick N Computer-aided classification of mammographic masses and normal tissue: linear discriminant analysis in texture feature space Physics in medicine and biology 1995 12 857 7652012 10.1088/0031-9155/40/5/010

[B20] Eltonsy NH Tourassi GD Elmaghraby AS A concentric morphology model for the detection of masses in mammography Medical Imaging, IEEE Transactions on 2007 12 880 889 10.1109/TMI.2007.895460 17679338

[B21] Metz CE Receiver operating characteristic analysis: a tool for the quantitative evaluation of observer performance and imaging systems Journal of the American College of Radiology 2006 12 413 422 17412096 10.1016/j.jacr.2006.02.021

[B22] Chang C-C Lin C-J LIBSVM: a library for support vector machines ACM Transactions on Intelligent Systems and Technology (TIST) 2011 12 27

[B23] Ojala T Pietikainen M Maenpaa T Multiresolution gray-scale and rotation invariant texture classification with local binary patterns Pattern Analysis and Machine Intelligence, IEEE Transactions on 2002 12 971 987

[B24] Choi JY Kim DH Choi SH Ro YM Multiresolution Local Binary Pattern texture analysis for false positive reduction in computerized detection of breast masses on mammograms SPIE Medical Imaging 2012 83152B 83157 10.1088/0031-9155/57/21/7029 23053352

[B25] Choi JY Ro YM Multiresolution local binary pattern texture analysis combined with variable selection for application to false-positive reduction in computer-aided detection of breast masses on mammograms Physics in medicine and biology 2012 12 7029 23053352 10.1088/0031-9155/57/21/7029

[B26] Haralick RM Shanmugam K Dinstein IH Textural features for image classification Systems, Man and Cybernetics, IEEE Transactions on 1973 610 621

[B27] Galloway MM Texture analysis using gray level run lengths Computer graphics and image processing 1975 12 172 179

[B28] Weszka JS Dyer CR Rosenfeld A A comparative study of texture measures for terrain classification Systems, Man and Cybernetics, IEEE Transactions on 1976 269 285

[B29] Kilday J Palmieri F Fox MD Classifying mammographic lesions using computerized image analysis Medical Imaging, IEEE Transactions on 1993 12 664 669 10.1109/42.251116 18218460

[B30] Kim DH Choi JY Ro YM Region based stellate features for classification of mammographic spiculated lesions in computer-aided detection Image Processing (ICIP), 19th IEEE International Conference on 2012 IEEE 2821 2824

